# Testing convergence among attachment methods: Adult Attachment Interview, Relationship-Specific Attachment Scale, and Implicit Association Test

**DOI:** 10.3389/fpsyg.2025.1487056

**Published:** 2025-04-08

**Authors:** Katja Petrowski, Roland Imhoff, Bjarne Schmalbach, Bernhard Strauß, Rainer Banse

**Affiliations:** ^1^Department of Internal Medicine III, University Medical Center Carl Gustav Carus at the University of Dresden, Dresden, Germany; ^2^Medical Psychology and Medical Sociology, University Medical Center of the Johannes Gutenberg University, Mainz, Germany; ^3^Department of Psychology, Johannes Gutenberg University, Mainz, Germany; ^4^Institute of Psychosocial Medicine and Psychotherapy, University Hospital Jena, Jena, Germany; ^5^Department of Psychology, University of Bonn, Bonn, Germany

**Keywords:** attachment, implicit association test, adult attachment interview, convergence, Relationship-Specific Attachment

## Abstract

Attachment theory describes the framework of how individuals relate in intimate relationships, including their confidence in receiving support and comfort from attachment figures. Three primary research directions, which have led to different methods, are used to investigate the attachment concept: developmental psychology, personality and socio-psychological research, and cognitive psychology. Conceptual differences and the need for convergence among these methods are of high interest to researchers. The present study explores convergence across different research traditions. The study involved 318 participants, including clinical and non-clinical groups matched for age and gender. Various psychometric instruments, including the Adult Attachment Interview (AAI), Relationship-Specific Attachment Scale (BBE), and Mother-Implicit Association Test (IAT), were used to measure attachment and related constructs. The hypothesized associations were investigated by structural equation models. Small correlations among the different methods were found, with stronger associations in the patient sample compared to that of the healthy individuals. The latent correlation was significant and small. Model fit indices indicated a much better fit in healthy controls. Even though there were moderate correlations among the different methods, the associations were generally stronger in the patient sample compared to that of the healthy individuals, even though the instruments were developed based on healthy individuals. The model fit was generally good in the healthy sample but less so in the patient sample. Due to the large differences in the theoretical and developmental background of the methods, it can be proposed that attachment representations may vary depending on the sample and method used.

## 1 Introduction

John Bowlby’s attachment theory emphasizes the deep emotional bonds that form between children and their caregivers and which shape the child’s later social, emotional, and cognitive development. This emotional bond influences a person’s characteristic ways of relating in intimate relationships with “attachment figures,” often one’s parents and/or romantic partners. At the heart of this theory is the inner working model, which refers to the mental representations of self, others, and relationships that are shaped by these early attachment experiences. These internal models guide how individuals approach relationships throughout their lives based on one’s confidence in the availability of the attachment figure as a secure base from which one can freely explore the world in times of no distress and seek support, protection, and comfort in times of distress. Therefore, the attachment theory is based on the effects of sensitivity of the attachment figure as well as separation, loss, and physical and sexual abuse experiences during the developmental course onto later interpersonal relationships.

Based on the inner working models, attachment typologies or dimensions can be observed in the behavior, which categorize attachment patterns into secure, anxious, avoidant, or disorganized types. Secure attachment is marked by trust and comfort with intimacy, while anxious attachment involves insecurity and fear of abandonment. Avoidant attachment is characterized by emotional distance and self-reliance, and disorganized attachment reflects a lack of clear strategy for dealing with attachment needs, often resulting from inconsistent caregiving. The attachment behavioral system is a dynamic system that drives individuals to seek closeness and security from attachment figures, particularly in times of distress, and it adapts based on experiences with caregivers. These systems work together to influence how individuals form and maintain relationships across their lifespan.

The attachment theory was applied to psychological phenomena in diverse psychological subdisciplines (developmental, personality, and cognitive psychology) with different theoretical ideas and methodical approaches (interview vs. questionnaires vs. implicit association test). Given its centrality in so many domains, it is of the utmost importance to obtain a good understanding of the construct and the measured facets of attachment.

The theoretical concept of attachment and the measured aspects of the different research tradition can be described as follows: Development-psychological research groups primarily examined the cognitive-emotional processing of attachment experiences in adults (states of mind with respect to attachment) and their influence on parent–child interaction. Primarily the Adult Attachment Interview (George et al., 1985), which measures the unconscious attachment representation of the interviewed person, marked this research direction.

In the personality and socio-psychological research tradition, studies focus on romantic relationships between adults. Hazan and Shaver postulated that the attachment typology derived from the mother–child relationship could be transferred to romantic adult relationships and recorded attachment-type-related behavior and ways of experiencing partnerships via self-assessments (questionnaires) (Hazan and Shaver, 1987).

In the cognitive research tradition, indirect access to the implicit relationship schema is gained by the latency-based Implicit Association Test (Greenwald et al., 1998). Given an automatic association of the partner with positivity, the compatible block (positive word, partner) should be easier to respond to, i.e., the responses thus being more immediate and less prone to error than to the incompatible block (negative word, partner). Individual differences in the magnitude of this effect can then be interpreted as indicators of how positive the partner’s schema is (Banse et al., 2013; Banse and Kowalick, 2007; Zayas and Shoda, 2005).

There are conceptual differences that need to be distinguished among the three research directions:

(1) The AAI foremost measures the unconscious aspects of the attachment experience and their processing via language analyses, whose defense processes and idealizations are taken into consideration (George et al., 1985). (2) The questionnaire measures attachment-relevant feelings, behaviors, and expectations on a consciously reflected level. (3) The IAT evaluates implicit relationship schemata, the effect of which the individual is unaware of (impact awareness). Besides the differences, there are also conceptual similarities between the developmental and the cognitive research tradition, measuring both the unconscious and unaware aspects of attachment. Even though there is no theory including the unaware implicit attachment schemata and the unconscious attachment representation, both shape how individuals perceive and interact in relationships based on unconscious mental frameworks or patterns that individuals develop based on early attachment experiences, particularly with primary caregivers. The implicit schemas influence how people interpret and respond to relational cues throughout their lives. Implicit schemata often operate automatically, influencing the way we react to relationship dynamics without conscious awareness (Banse et al., 2013; Banse and Kowalick, 2007; Zayas and Shoda, 2005).

However, the unconscious attachment representations influence how individuals reflect and discuss their attachment childhood experiences with their primary caregivers, specifically their feelings, memories, and the quality of their relationships. The unconscious attachment representations are not explicitly articulated but can be inferred from how they narrate their attachment history by analyzing the coherence, consistency, and emotional tone of the responses (George et al., 1985). Therefore, the measured aspect of attachment with the used methods is crucial for a full understanding of the construct (Strauss et al., 2022).

With the growing number of available methods and empirical results on the different facets of attachment, an intense discussion was launched in regard to the convergence of the individual instruments (Strauss et al., 2022). In addition, the empirical convergences of the individual instruments from the various research traditions had only been small (Crowell et al., 1999; Crowell and Treboux, 1995; Stein et al., 2002). Fraley et al. (2000) showed that attachment anxiety and avoidance form important dimensions underlying the self-report questionnaires with a high convergence. Concerning categorical attachment measures, Bartholomew and Shaver (1998) showed that the convergence was only moderate. The Implicit Association Test has only been investigated in combination with self-report questionnaires, the convergence of which was moderate (Banse et al., 2013), but not in reference to an interview method. The convergence between self-report and interview methods was in general quite low (Crowell et al., 2016; Roisman et al., 2007; Shaver and Mikulincer, 2005; Strauss et al., 2022). Thus, convergence is higher in methods of the same research tradition compared to different research traditions.

This lack of convergence might be explained from a methodological point of view: Measures of adult attachment differ in terms of domain (family, peer, or romantic relationship), method (interview, Q-Sort, self-report), and dimensionality (categories, prototype ratings, or dimensions) (Bartholomew and Shaver, 1998). Assuming that all the methods of adult attachment research refer to a theoretical construct (Bartholomew and Shaver, 1998), it should be possible to place them on a continuum:: one pole of the continuum consists of elaborate outside evaluations, which grasp the unconscious aspects of the attachment and generalized attachment presentations; the other pole consists of self-assessment questionnaires that measure attachment-relevant feelings, behaviors, and expectations about the mostly specified reference focuses (time, attachment figure).

The position according to which the conception and the inquiry of generalized or specific attachment representations do not constitute an antagonism, which was represented by Collins and Read (1994), who correlated generalized and specific attachment representations in the model of a hierarchical cognitive network. In such a network, attachment patterns of adults consist of a league of individual attachment concepts correlated among themselves as organized according to the degree of abstraction, becoming more concrete in descending order (e.g., general model of self/ others, model of father-son relationship, model of own relationship to father). Thereby, models with a higher degree of abstraction are applied to a broad spectrum of relationships and situations at the expense of the specific fit, whereas more concrete models allow for more precise fits, however, only for a limited realm of relationships/ situations. According to the concept of the hierarchical network of attachment models, individual attachment models do not only differ in reference to the characteristic of individual network components (models if attachment/ relationships) but also in regard to the connection structure of the network, for example, in the form of correlations among the individual models. Generally, in cognitive psychology, networks are understood to be more complex and more adaptive, with the more specific (heterogeneous) models marked with high concreteness (e.g., positive model of father versus negative model of mother). In the realm of attachment research, this can only be regarded in a restricted sense, since defense and idealization processes might lead to specific but not differentiated attachment models (Collins and Read, 1994).

Over the past decade, attachment theory was assessed with different methods measuring different facets of the attachment construct. It is very important to obtain a good understanding of the different facets measured and how they relate to each other in order to understand the full body of the attachment constructs. The least empirical results are published on the convergence between the development-psychological research tradition and the cognitive research tradition. Comparisons of attachment facets that are more indirect or unaware, such as implicit schemata and unconscious attachment representations, are still lacking. To our knowledge, there is not yet a theory that combines the indirect, unaware implicit schemata with the unconscious attachment representations. The Implicit Association Test has not yet been investigated in reference to an interview method, such as the Adult Attachment Interview. Both approaches measure unaware or unconscious processes that shape how individuals perceive and interact in relationships based on early attachment experiences, particularly with primary caregivers. However, implicit schemata operate automatically, influencing the way we react to relationship dynamics (measured with the Implicit Association Test in reaction time). In contrast, the unconscious attachment representations influence how individuals reflect and discuss their attachment childhood experiences, with the coherence, consistency, and emotional tone of the responses being measured (with the Adult attachment Interview in linguistic analyses). To the best of our knowledge, the convergence between implicit unaware schemata and unconscious attachment representation measured with these two methods is still unknown.

In order to study implicit unaware schemata and unconscious attachment representation, it has to be considered that unconscious attachment representations are most measurable in the unresolved/disorganized attachment classification during the Adult Attachment Interview. Individuals in this classification tend to display contradictory, incoherent, or fragmented narratives when discussing past attachment experiences, particularly those related to trauma, loss, or abuse (George et al., 1985). In contrast, individuals with secure-autonomous, dismissive-avoidant, or preoccupied attachment classifications, while still reflecting on attachment experiences, generally present more coherent and organized attachment representations, making their unconscious attachment patterns less readily measurable (George et al., 1985). These unconscious unresolved/disorganized attachment representations are predominantly shown in clinical samples with anxiety disorders (Dozier et al., 2008). In order to investigate the convergence between unconscious attachment representation and implicit unaware schemata, these unconscious attachment representations have to be measurable, and are currently only highly readily measurable in clinical samples with a homogeneous anxiety disorder (Dozier et al., 2008). By including clinical and non-clinical samples, all the attachment classifications are included in the comparisons, especially the unresolved/disorganized attachment classifications. In addition, the measures were either developed and their convergence tested in healthy or clinical samples (e.g., Schützmann, 2004; Crowell et al., 1999). Further exploration of conscious cognitive processed versus unaware and unconscious attachment facets have to be explored in homogenous clinical and non-clinical individuals, such as patients with panic disorders and/or agoraphobia (based on Dozier et al., 2008).

The Adult Attachment Interview and the Implicit Association Test both measure unaware or unconscious processes that shape how individuals perceive and interact in relationships based on early attachment experiences, particularly with primary caregivers. Since in the clinical and non-clinical sample unconscious attachment representations are highly measurable, both measures can detect unaware or unconscious processes and, therefore, a higher convergence might be visible.

Self-report questionnaires measure cognitive processed, conscious attachment-relevant feelings, behaviors, and expectations about the specified attachment figure (Bartholomew and Shaver, 1998). Since the conscious facets of attachment in contrast to the unconscious facets should be included to differentiate them from the unaware implicit unconscious facets of attachment, the self-report questionnaire (the Relationship Specific Questionnaire) was chosen to measure the whole body of attachment construct. Due to the higher percentage of unconscious attachment representation in the clinical sample and organized/conscious attachment representation in the healthy sample, the comparison might help to better understand the convergence of the concepts/methods used. The Implicit Association Test already showed a moderate convergence with self-report questionnaires (Banse et al., 2013), so it can be assumed that the Implicit Association Test might evaluate both conscious and unconscious facets of attachment and the replication of the convergent association can be hypothesized.

In order to specify the effect of the different methods, they were modeled separately as latent variables in a healthy as well as anxiety patient sample. In sum, the present research aims to tap into adults’ attachment representations with a measurement battery consisting of methods particularly prominently used in clinical (interview), personality (questionnaires), and social (implicit association tests) psychology and explore their convergent validity of conscious and unconscious facets of attachment.

## 2 Methods

### 2.1 Participants

For this study, *N* = 318 subjects were recruited. The participants were 36 years of age on average (*SD* = 11.2, *Min* = 18, *Max =* 65). Two-thirds of the participants were women (66%) and 76% had a steady partner; 42% of the participants had graduated with a 10th grade education from a secondary school with vocational training. The clinical sample comprised *n* = 175 participants, the non-clinical sample *n* = 143. With regard to age and gender, no significant differences showed up, suggesting that matching had been successful. However, partner situation (*p* = 0.028) and education (senior high school graduation with *p* < 0.001, but not 10th grade education from a secondary school with vocational training with *p* = 0.606) showed significant differences. In the clinical sample, over 80% of the subjects had a steady partner in comparison to the healthy individuals (nearly 70%). With regard to education, 64% of the patients had not graduated from senior high school as opposed to nearly 49% of the healthy individuals. The significant differences in terms of inability to work (*p* < 0.001) were as expected: only 5% of the healthy subjects were unable to work compared to 47% of the patients. The exact values and additional variables are presented in Table 1.

**Table 1:** Correlation matrix.

	1	2	3	4	5	6
Total sample (*n* = 286)						
1—IAT odd	1	0.720^***^	0.214^***^	0.168^**^	0.135^*^	0.025
2—IAT even		1	0.242^***^	0.215^***^	0.184^**^	0.020
3—BBE secure			1	0.469^***^	0.087	0.371^***^
4—BBE dependent				1	0.050	0.091
5 AAI—Waters Scale 1					1	0.047
6 AAI—Waters Scale 2						1
Patient sample (*n* = 145)						
1—IAT odd	1	0.756^***^	0.265^**^	0.189^*^	0.120	0.070
2—IAT even		1	0.327^***^	0.267^**^	0.133	0.067
3—BBE secure			1	0.399^***^	−0.054	0.393^***^
4—BBE dependent				1	−0.087	−0.038
5 AAI—Waters Scale 1					1	−0.002
6 AAI—Waters Scale 2						1
Healthy sample (*n* = 141)						
1—IAT odd	1	0.720^***^	0.214^**^	0.168^*^	0.135	0.025
2—IAT even		1	0.242^**^	0.215^**^	0.184^*^	0.020
3—BBE secure			1	0.469^***^	0.087	0.371^***^
4—BBE dependent				1	0.050	0.091
5 AAI—Waters Scale 1					1	0.047
6 AAI—Waters Scale 2						1

IAT, Implicit Association Test; BBE, Relationship-specific binding scales; AAI, Adult Attachment Inventory. ^*^Significant at the 0.05 level; ^**^Significant at the 0.01 level; ^***^Significant at the 0.001 level.

### 2.2 Procedure

The study data was obtained from a collaborative study between the Institute of Psychosocial Medicine and Psychotherapy at the University Hospital Jena and the Clinic for Psychotherapy and Psychosomatics at the University Hospital Carl-Gustav-Carus at the TU Dresden. The data collection took place between October 2011 and April 2014. For the clinical sample, patients were recruited from an outpatient day clinic and an inpatient setting. The healthy sample was recruited according to matching age, gender, and educational level. To obtain a very homogeneous sample of patients, only patients between 18 and 65 years of age with a primary panic disorder with or without agoraphobia or with agoraphobia without panic disorder in their history (ICD-10 diagnoses F40.01, F40.1, F41.0) were included in the study. Patients were also permitted to have a secondary diagnosis of major depression, dysthymia, alcohol/drug abuse, social phobia, or other specific phobias; however, these diagnoses should not be the primary treatment concern. The lifetime presence of other mental disorders (Axis I) or personality disorders (axis II) led to exclusion from the study.

Most of the patients (69%) showed a panic disorder with agoraphobia (F40.01), 29% displayed a panic disorder only (F41.0), and 2% exclusively agoraphobia without panic attacks (F40.00). Most of the patients showed at least one comorbidity (56%), with 49% of patients comorbid with an affective disorder, 22% with a specific phobia, 12% with a social phobia, and 3% with alcohol abuse.

The healthy individuals who met the criteria of any current or lifetime axis I or axis II disorder were excluded from the study. The healthy sample were significantly different from the patients in the Anxiety- Phobia Scale and in the GSI of the Symptom Check List (Franke, 2000) as well as the Beck Depression Inventory (*p* < 0.001) (Hautzinger et al., 1995).

All individuals (patients and controls) needed to have experienced a close relationship with one person in their life but did not necessarily have to have a partner at the present time. All subjects were required to take part in the Structured Clinical Interview for DSM-IV (SCID) (Wittchen et al., 1997) to check for the Axis I and Axis II disorders based on the criteria of the fourth edition of the Diagnostic and Statistical Manual of Mental Disorders (DSM-IV) (Saß et al., 2003).

All subjects gave their written informed consent. Healthy individuals as well as patients underwent the same diagnostic procedure with the attachment measurement (all measurements at 1 day in the same order). First the latency-based measure, the IAT, and afterwards the Adult Attachment Interview were implemented in this clinical and non-clinical sample (Strauss et al., 2022). The study was performed in agreement with the Declaration of Helsinki and has been approved by the University of Dresden Medical Faculty Ethics Review Board (EK 79032011).

### 2.3 Instruments

The Adult Attachment Interview (George et al., 1985), a semi-structured interview with 18 questions, detects the current representation of attachment experiences regarding the past and present (primary attachment figure, mother, father). To evaluate the unconscious aspects of the inner working model (= mental organization of attachment), the responses of the interviewee were analyzed using verbatim transcripts. The coding process refers to linguistic aspects, such as the degree of coherence. Based on the content analysis, the subjects can be classified into four patterns of attachment representation (Main et al., 2003): Secure/autonomous attachment (F), insecure/preoccupied (E), insecure/dismissing (Ds), and disorganized/unresolved trauma (U). Secure (F) and insecure (Ds and E) provide the organized patterns, while the unresolved attachment (U) is the so-called disorganized pattern. The AAI has very good psychometric properties, showing high test–retest reliability for the three main classifications with 78–90% and *κ* = 0.63 to κ = 0.79 and high interjudge reliabilities (Bakermans-Kranenburg and van IJzendoorn, 1993; Sagi et al., 1994). In the present study, the interrater reliability of the reliable certified judges (J.B./K.B. and K.N.) was 59% based on 10% of the AAIs for the four attachment representations F, Ds, E, and U (κ = 0.43, *p* < 0.001). It is known from studies using the AAI that the interrater reliability is sometimes not optimal, which seems acceptable on a group level but should be avoided on an individual level if, e.g., AAI results are used for forensic purposes (cf. Van IJzendoorn and Bakermans-Kranenburg, 2021).

Since a dimensional approach is more easily comparable to the other attachment methods, the two-dimensional scales “security versus insecurity” and “dismissing versus preoccupied” developed by Waters et al. (2018) were used in the present study. Waters et al. (2018) applied a discriminant analysis to more than 500 AAIs and derived discriminant weights for scoring “secure vs. insecure” and “dismissing vs. preoccupied.” In reference to the standardized values, a score higher than 0.00 on the secure vs. insecure scale represents a secure attachment orientation. A score higher than 0.01 on the dismissing vs. preoccupied scale represents a dismissing attachment orientation.

The Relationship-Specific Attachment Scale (BBE) (Asendorpf et al., 1997) describes the current attachment to the mother based on two dimensions: secure-anxious and dependent-independent. Item responses are recorded on 5-point Likert scales and aggregate scales; scores are calculated via summation. Both scales of this questionnaire show good reliabilities with internal consistencies from *α* = 0.71 to 0.87 and retest reliabilities from r = 0.70 to 0.86 (Asendorpf et al., 1997). The validity check revealed significant hypothesized correlations with Bartholomew’s bond prototypes, the results of a network questionnaire on important relationships, and a partnership satisfaction questionnaire. The relationship specificity was also tested using this questionnaire, as it is available in different forms for mother, father, partner, and friend. Apart from the mean correlations between the mother and father bond, there were low correlations (mean across all relationships –= 0.29) with regard to the different bond figures (Asendorpf et al., 1997).

The Mother-IAT (Banse and Kowalick, 2007) is constructed analogous to the standard IAT (Greenwald et al., 1998). It consists of three training blocks (1, 2, 4) and two measuring blocks (3, 5). The discrimination tasks with positive vs. negative (attributes, 40 repetitions) and mother vs. stranger (target concept, 160 repetitions) were presented to the subjects. For the positive–negative-discrimination, there are four positive and four negative attributes. For the mother-stranger-discrimination, four individual items for the mother and four items that are not characteristic of the partner/mother were selected in advance. The sequence of blocks and the randomized presentation of items is constant. As total score, the D-score, as proposed by Greenwald et al. (1998), was used (see there for calculation rules). More positive scores indicate more positive implicit attitudes toward the mother. The reliability (internal consistency) was satisfactory with *α* = 0.83 for mother (Zimmermann and Maier, 2002). In the present study, the internal consistency was satisfactory as well with α = 0.81 for mother. A satisfactory test–retest reliability (partner: *r* = 0.65 to 0.69; mother: *r* = 0.51) has been shown (Zayas and Shoda, 2005; Zimmermann and Maier, 2002). In addition, significant positive correlations between the response times and the secure attachment scale of the Relationship Questionnaire could be demonstrated (RQ) (Bartholomew and Horowitz, 1991) as well as negative correlations with the subscales avoidance and anxiety (Banse and Kowalick, 2007; Zayas and Shoda, 2005).

The psychopathological burden and the severity of the panic-agoraphobia symptoms were measured by the following instruments: (1) the Symptom-Check-List (SCL) (Derogatis and Melisaratos, 1983; Franke, 2000), consisting of 90 items with a five-point rating scale (range: 20–80) to evaluate psychological and physical impairment (General Symptom Index, GSI; Positive Symptom Total, PST); and (2) the Beck-Depression-Inventory II (BDI) (Beck et al., 1961; Hautzinger et al., 1995) to evaluate the depressive symptoms, which consists of 21 symptoms rated in terms of intensity from 0 to 3 (range: 0–63).

### 2.4 Statistical procedure

**Figure 1:**
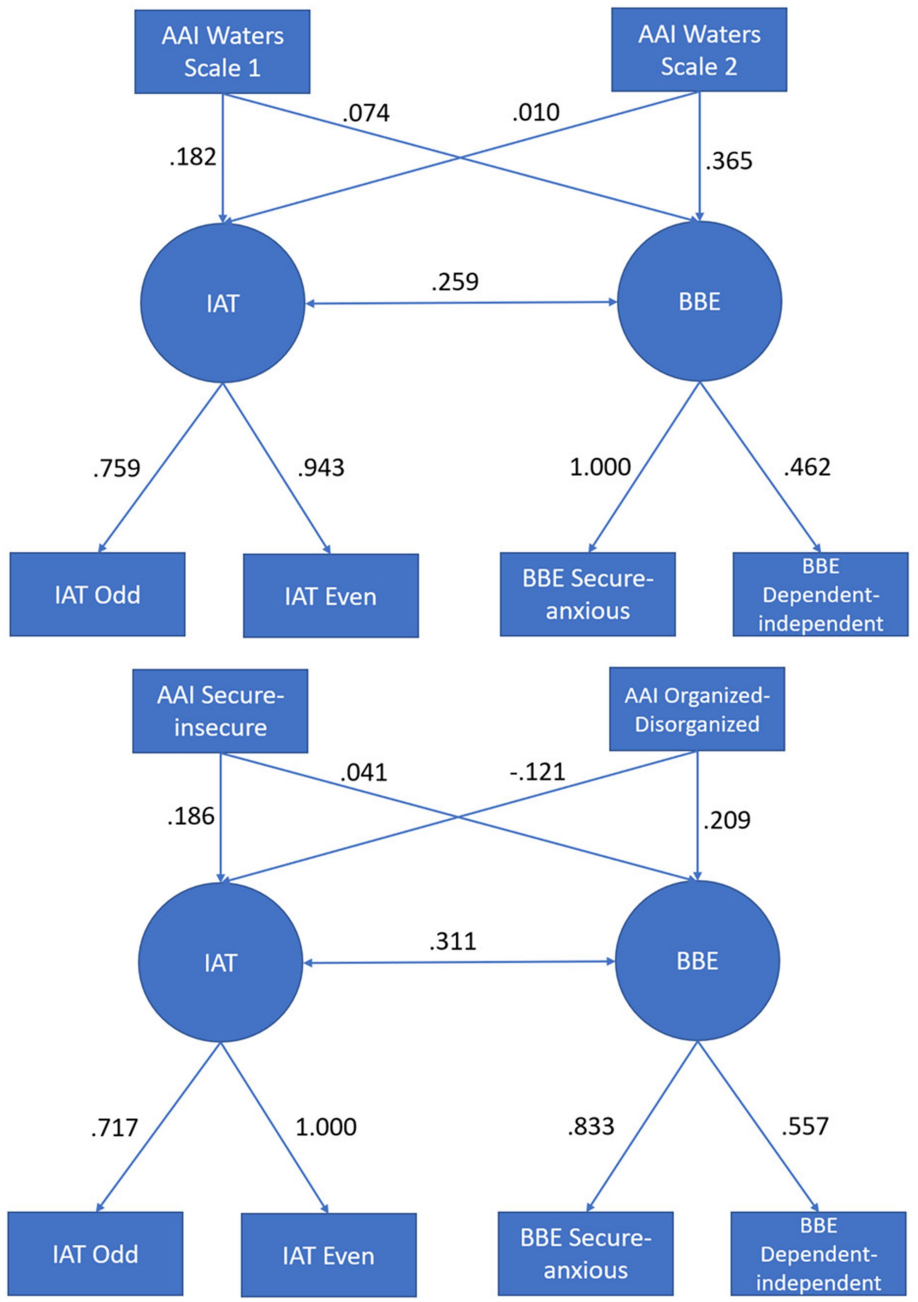
The tested measurement models with standardized parameter estimates. IAT, Implicit Association Test; BBE, Relationship-specific binding scales; AAI, Adult Attachment Interview; odd, odd IAT trials; even, even IAT trials.

The statistical analyses were conducted in R, using the packages lavaan, psych, and semTools (Jorgensen et al., 2019; Revelle, 2024; Rosseel, 2012). Of the 318 initial participants, 286 were complete observations and were included in the analysis. Initially, we conducted parallel analysis to ascertain the number of latent factors and ran exploratory factor analysis to get a elementary understanding of the loading pattern. To then analyze the latent correlations between the instruments of interest, we tested a measurement model in which variables were included as interval scales at the aggregate level. Specifically, a latent IAT factor is comprised by odd and even IAT trials, while a latent BBE factor is measured by the secure and dependent BBE subscales, as well as the Waters Scales 1 and 2 independently (see Figure 1). In a second model, the AAI Waters Scales were replaced by the AAI- Categories secure-insecure and organized-disorganized. In addition, the variance of the latent variables was fixed to 1 for identification purposes (Little et al., 2006). We estimated the model using robust maximum likelihood estimation (Satorra and Bentler, 2001) and referred to the commonly used fit indices and cutoff values (Hu and Bentler, 1999; Schermelleh-Engel et al., 2003): A non-significant χ^2^, Comparative Fit Index (*CFI*), and Tucker-Lewis Index (*TLI*) greater than 0.950 for acceptable and greater than 0.970 for good fit, a Root Mean Square Error of Approximation (*RMSEA*) smaller than 0.080 for acceptable and smaller than 0.050 for good fit, and a Standardized Root Mean Square Residual (*SRMR*) smaller than 0.100 for acceptable and smaller than 0.050 for good fit were used.

## 3 Results

In Table 1, we present a zero-order correlation matrix among all attachment measures. The correlations range between 0.020 and 0.371. Observed correlations between the composite scores for the three instruments were *r*_IAT,BBE_ = 0.267, *r*_IAT,AAI_ = 0.133, and *r*_BBE,AAI_ = 0.260.

We then conducted parallel analysis, yielding strong evidence of three substantial factors in the data at hand, with empirical eigenvalues of 2.10, 1.44, and 1.14 versus 1.29, 1.16, and 1.07 for the 95th percentile of the simulated eigenvalues. The subsequent exploratory factor analysis yielded loadings between 0.451 and 0.994 across the three factors.

Based on these preliminary findings, we tested the same correlations but modeled the constructs as latent factors. It became evident that the AAI with the Waters scales cannot be modeled appropriately because its discriminant analytically driven components do not overlap sufficiently for them to represent a singular latent factor. To remedy this, we tested models that included manifest variables instead. Model 1 used the Waters Scales 1 and 2 (see Figure 1) and Model 2 the AAI binary categorical variables (secure vs. insecure and disorganized vs. organized).

The initial fit for the model laid out in Figure 1 was acceptable, with a non-significant χ^2^-test and exceptional fit indices (see Table 2). However, the negative residual variance of the BBE secure indicator (*θ* = −0.072, *SE* = 0.251, *p* = 0.773) calls into question the interpretability of this model. Since the variance was not significantly different from zero as per its confidence interval (Kolenikov and Bollen, 2012), we fixed it to 0 in order to make the model interpretable, resulting in minor improvements for Model 1a, which can be considered for all intents and purposes as the same model. For the estimation of the (semi-)latent correlations, we referred to the latter model.

**Table 2:** Model fit statistics.

Model	χ^2^ (*df*)	*p*	CFI	TLI	RMSEA	SRMR
Total sample (*n* = 286)						
1	6.087 (5)	0.298	0.996	0.990	0.029	0.035
1a	6.041 (6)	0.419	1	1	0.005	0.033
2	6.369 (5)	0.272	0.996	0.988	0.030	0.026
2a	5.969 (6)	0.427	1	1	0	0.024
Patient sample (*n* = 145)						
1	12.908 (5)		0.962	0.895	0.100	0.062
1a	11.366 (6)		0.969	0.928	0.083	0.057
2	16.652 (5)		0.955	0.873	0.104	0.054
2a	13.029 (6)		0.962	0.912	0.087	0.043
Healthy sample (*n* = 141)						
1	1.312 (5)		1	1.088	0	0.018
2	1.277 (6)		1	1.098	0	0.018

CFI, Comparative Fit Index; TLI, Tucker-Lewis Index; RMSEA, Root Mean Square Error of Approximation; SRMR, Standardized Rot Mean Square Residual; 1a, error variance of BBE secure indicator set to 0; 2a, error variance of IAT even trial indicator set to 0.

The alternative Model 2—including the AAI categories—evinced a similar fit to the data (see Table 2). However, we again encountered negative error variances, this time for the IAT even trial indicator (θ = −0.016, *SE* = 0.059, *p* = 0.788). Since it was again not significantly different from 0, we fixed it to 0 to gain an interpretable model (Model 2a), with a virtually unchanged fit. We again estimated the (semi-)latent correlations using the last model configuration.

We then applied the same models to the patient and healthy control subsamples. For the patients, model fit was substantially worse than for the healthy individuals. Furthermore, we had to apply the residual variance respecifications only for the patient subsample (Model 1: θ = −0.401, *SE* = 0.978, *p* = 0.682; Model 2: θ = −0.064, *SE* = 0.133, *p* = 0.630). For the healthy participants, both models were valid. Latent correlations for the subsamples can be found in Table 3.

**Table 3:** Latent correlations (Model 1a and 2a).

	Model 1a				Model 2a			
	1	2	3	4	1	2	3	4
Total sample (*n* = 286)								
1 IAT	1	0.259^***^	0.182^**^	0.010	1	0.311^***^	0.186^**^	0.121^*^
2 BBE		1	0.074	0.365^***^		1	0.041	−0.209^**^
3 AAI—Waters Scale 1			1	0.049			1	−0.475^***^
4 AAI—Waters Scale 2				1				1
Patient sample (*n* = 145)								
1 IAT	1	0.351^***^	0.140	0.072	1	0.449^***^	0.078	0.046
2 BBE		1	−0.053	0.393^***^		1	−0.165^*^	−0.238^**^
3 AAI—Waters Scale 1			1	−0.002			1	−0.390^***^
4 AAI—Waters Scale 2				1				1
Healthy sample (*n* = 141)								
1 IAT	1	0.200^*^	0.170^*^	−0.089	1	0.200^*^	0.278^***^	0.237^**^
2 BBE		1	0.188^*^	0.364^***^		1	0.176^*^	−0.179^*^
3 AAI—Waters Scale 1			1	0.041			1	−0.543^***^
4 AAI—Waters Scale 2				1				1

Significant at * = 0.05, ** = 0.01, *** = 0.001.

To test for equality between groups of the latent correlations/regression terms, we further tested and compared the models: (1) with no between-group constraints and (2) with these parameters constrained to be equal between the two subsamples. For Model 1, we found no significant changes in model fit by adding these constraints: Δχ^2^(5) = 6.50, *p* = 0.261, Δ*CFI* = 0.005, Δ*TLI* = 0.006, Δ*RMSEA* = 0.007, Δ*SRMR* = 0.020. Only *SRMR* was elevated somewhat.

Similarly for Model 2, there were only marginal changes in model fit caused by constraining correlations/regressions to be equivalent between groups: Δχ^2^(5) = 5.94, *p* = 0.312, Δ*CFI* = 0.004, Δ*TLI* = 0.001, Δ*RMSEA* = 0.001, Δ*SRMR* = 0.025. Again, *SRMR* was the sole outlier.

## 4 Discussion

### 4.1 Convergence of attachment constructs

The attachment theory is of importance in many developmental, social, personality, and clinical domains. The different psychological areas (developmental, personality, cognitive, psychology) lead to different methodical approaches (interview vs. questionnaires vs. implicit association test) developed based on healthy individuals or patient samples. Since all the methods propose to measure attachment, it is very important to obtain a good understanding of the constructs measured and how the concepts are related to each other.

In order to compare the measured constructs, the temporal reference, the attachment figure mother, as well as the dimensional data format was standardized and only the methodology differed. Based on the dimensional measurement, the correlations between the three different methods were low to moderate. Hereby, the BBE-secure shows the highest correlation with *r* = 0.37–0.39 with the AAI Waters scale. The moderate correlations between the AAI-Waters scales and questionnaire data are in line with the literature (Crowell and Waters, 2006; Glenn, 2018; De Haas et al., 1994; Waters et al., 2018). Even though different questionnaires were used, the associations were similar. However, the low associations between the questionnaires and the IAT (*r* = 0.21–0.33) is in contrast to previous results from the literature (Robinson et al., 2021). It is astonishing that the associations are generally higher in the patient sample than in the healthy individual or total sample. This is remarkable since all the instruments were not developed based on patients but rather based on healthy individuals. However, participants in clinical settings are more likely to have reflected on attachment-related patterns through therapeutic experiences, which could influence their responses on attachment measures. In addition, it might help that the variance of the facets of attachment representations might be higher in clinical sample than in non-clinical sample.

As a next step, the latent model was calculated in order to eliminate possible measurement errors. In these models, the model fit was very good in the healthy sample. The high model fit is explicable since all three methods were developed on a healthy sample as well as measuring the same domain and dimensionality. Herein, the Waters scales secure-insecure and the BEE_scale secure converge highly. The Waters scale dismissing-preoccupied shows covariances with the IAT_odds and even converge with the BBE secure.

For the patients or the combined sample, the model fit seemed to be good as well, however, there were still negative error variances. This might be explained by having been developed based on a healthy sample with a predominantly secure and organized attachment representation. A predominantly insecure or disorganized attachment representation was even found in patients with earlier anxiety disorders (Dozier et al., 2008). An additional argument for the lack of model fit might be the high heterogeneity of the indicators, especially for the AAI. This might be explained by the development process of the interview, which was not based on test statistical procedures (secure-insecure, preoccupied-dismissing). In addition, the Waters scales were built on a discriminate analysis using the same ratings for both scales. Furthermore, the underlying model of the BBE does not show high model fit and the proposed model was not replicated by a CFA (Asendorpf et al., 1997). Therefore, when the model fits of the single models are not high, then the combined model is not high either.

Concerning the differences between healthy individuals and the clinical sample, we tested for invariance of latent correlations and regressions and found no meaningful differences. Taken together with the excellent model fit when considering the subsamples individually, this is evidence that the convergence/divergence between the attachment measures is similar for both subsamples under consideration, clinical and healthy.

Although there is evidence in the literature indicating a limited overlap between self-report-based and interview measures (especially the Adult Attachment Interview, AAI, e.g., Roisman et al., 2007), there is still some evidence missing concerning the implicit association test based on a cognitive psychological attachment concept that further underlines the necessity of further clarification (see also Duschinsky, 2021). If all the measures would be related to exactly one construct, high convergence could be expected. We know from previous research and from the present findings that this is not the case (e.g., Roisman et al., 2007). Divergence of the measures might have different explanations. The source of the data (self-report vs. observer rating) might cause divergence due to a reflection of defensive process (e.g., idealization) or memory bias in self-reports or biases related to expectations or the influence of the observer’s attachment in observer ratings. The highest divergence might be expected when measures are compared that relate to different time domains (present vs. past), referents (mother/father vs. partner or therapist), or data format (dimensions vs. categories), which was harmonized in the present study (cf., Cassidy and Shaver, 2016). Interview and self-report might differentially activate the attachment system, especially in an interview situation with a strange person who is asking questions that are supposed to “surprise the unconscious,” as is the case for the Adult Attachment Interview (AAI) (George et al., 1996).

In the IAT, given an automatic association of the partner with positivity, the responses are thus more immediate and less prone to error than to the incompatible block. Individual differences in the magnitude of this effect can then be interpreted as indicators of a more indirect, implicit positive of the partner (Banse et al., 2013; Banse and Kowalick, 2007; Zayas and Shoda, 2005). Both unconscious and indirect implicit schemata or representations might be located more closely on the awareness dimension.

Therefore, after the standardization of the domain (family, peer, or romantic relationship) and the dimensionality (categories, prototype ratings, or dimensions; Bartholomew and Shaver, 1998), the variance due to the method fits the empirical model in a healthy sample. Bartholomew and Shaver (1998) proposed a continuum: The one pole of the continuum consists of elaborate outside evaluations, which grasp the unconscious aspects of the attachment and generalized attachment presentations; other pole contains self-assessment questionnaires, which measure attachment-relevant feelings, behaviors, and expectations.

However, the hierarchical cognitive network seems to be separate. Herein, the attachment patterns of adults organize themselves according to the degree of abstraction, becoming more concrete in descending order. These cognitive networks are understood to be more complex and more adaptive; the more specific (heterogeneous) models are marked with high concreteness (e.g., positive model of father versus negative model of mother). In the realm of attachment research, this can only be regarded in a restricted sense, since defense and idealization processes might lead to specificity but in improvement-worthy differentiated attachment models, especially for the clinical sample.

Based on the attachment-elephant analogy, measures of adult attachment are only partially convergent, since the methods measure all different aspects of the attachment construct, and it needs to be clarified which aspect of attachment is actually being assessed in individual clinical investigations. Therefore, it is of high interest to understand precisely which aspect of attachment is measured with which instrument in clinical as well as non-clinical samples. Based on this knowledge, studies should choose the instrument more carefully depending on the research question and not based on the applicability or time duration of the instruments. Since one might propose that the different measures are in fact related to one construct (Strauss et al., 2022), additional studies using different instruments out of different research traditions in clinical and non-clinical samples have to be conducted. Therefore, it would also be of interest whether differences in clinical diagnoses might influence these convergences in attachment representations. Based on this empirical evidence, the attachment theory could be specified more and take into account the different languages (unaware implicit attachment schemata, unconscious attachment representation) in integrated attachment theory.

### 4.2 Limitations

The present study’s significant strength lies in the substantial size of the two groups under investigation, especially in the context of complex clinical assessments. This robust sample size enables us to standardize variations in domains and dimensionality differentiated by Bartholomew and Shaver (1998). It should be noted that, in the context of estimating effect sizes, our sample is acceptable but not exceptional. The subsamples of ~150 observations are close to the point-of-stability identified by Schönbrodt and Perugini (2013).

Furthermore, a homogeneous clinical sample alongside a matched control group comprising non-clinical individuals forms a solid foundation for group comparisons and testing measure convergence. For the AAI, certified raters were enlisted to ensure procedural quality. However, the interrater reliability was not as high as we wished for. It is known from studies using the AAI that the interrater reliability is sometimes not optimal, which is acceptable on a group level but should be avoided on an individual level if, e.g., AAI results are used for forensic purposes (cf. Van IJzendoorn and Bakermans-Kranenburg, 2021). Nevertheless, the AAI analyses have to be replicated by two experienced raters. While the homogeneity of our clinical sample served to mitigate biases stemming from other psychopathological conditions, it is worth noting that our specific patient group, consisting of individuals with anxiety disorders (specifically, panic disorder/agoraphobia), may not necessarily represent other clinical populations.

## 5 Conclusion

The results showed low-to-moderate correlations among the different methods, with the highest correlation observed between BBE-secure and the AAI Waters scale. Surprisingly, the associations were generally stronger in the patient sample compared to that of the healthy individuals, even though the instruments were developed based on healthy individuals. Using latent models, the model fit was generally good in the healthy sample but less so in the patient sample. One of the strengths is the inclusion of the cognitive psychological attachment tradition with the implicit relationship schemata in combination with the Adult Attachment Interview from the developmental psychology tradition. With this approach, a first step to understand unconscious, implicit aspects of attachment was gained. Due to the large differences on account of the theoretical and developmental background of the methods, comparing attachment measures across different methods and populations is quite difficult and suggests that attachment representations may vary depending on the sample and method used.

## Data Availability

The raw data supporting the conclusions of this article will be made available by the authors, without undue reservation.
